# Is sleep apnea truly associated with hearing loss? A nationwide, population-based study with STOP-BANG questionnaire

**DOI:** 10.3389/fpubh.2023.1170470

**Published:** 2023-05-25

**Authors:** Jeon Mi Lee, Hyun Jin Lee

**Affiliations:** ^1^Department of Otorhinolaryngology, Ilsan Paik Hospital, Inje University College of Medicine, Goyang, Republic of Korea; ^2^Department of Otorhinolaryngology-Head and Neck Surgery, Incheon St. Mary’s Hospital, College of Medicine, The Catholic University of Korea, Seoul, Republic of Korea

**Keywords:** sleep apnea, hearing loss, STOP-Bang questionnaire, age, KNHANES

## Abstract

**Objectives:**

We aimed to investigate the effect of obstructive sleep apnea (OSA) on hearing ability.

**Methods:**

We retrospectively reviewed the population-based survey data collected by the Korean National Health and Nutrition Examination Survey between January 1, 2019 and December 31, 2020. The data included 3,575 participants who completed the STOP-BANG questionnaire (SBQ) and pure-tone audiometry. OSA risk was assessed using the SBQ, and the hearing level was compared between the risk groups.

**Results:**

Among the 3,575 participants, 2,152 (60.2%), 891 (24.9%), and 532 (14.9%) were classified as being low, intermediate, and high risk, respectively. The intermediate- and high-risk groups showed significantly worse hearing levels than the low-risk group. When age and sex were adjusted, the hearing level did not differ between the risk groups.

**Conclusion:**

The study found that the presence of OSA minimally affected hearing level. Because hearing loss due to hypoxic damage develops over a long period of time, further research on the association between the duration of OSA, rather than the presence or severity of OSA, and hearing loss is needed.

## Introduction

Obstructive sleep apnea (OSA) is a disorder that causes frequent pauses in breathing during sleep due to the repetitive collapse of the upper airway. OSA causes a reduction in oxygen in the blood (hypoxia) and repeated awakenings during sleep. This can injure the delicate hair cells responsible for hearing in the inner ear, resulting in hearing loss. Hypoxia-induced oxidative stress, inflammation, and alterations in blood flow to the inner ear are believed to contribute to the pathophysiological basis of hearing loss in OSA (1, 2). Additionally, the recurrent arousals and sleep fragmentation associated with OSA can disrupt the normal physiological processes involved in hearing, leading to further damage over time (3).

The prevalence of OSA varies greatly from 0.26 to 49.7% (4, 5), and approximately 80 to 90% of patients with OSA remain undiagnosed (6, 7). The difference in the prevalence among these studies is likely due to the difficulty in diagnosing OSA. The gold standard for diagnosing OSA is an overnight polysomnogram (PSG), which measures sleep apnea parameters, such as the respiratory disruption index or the apnea-hypopnea index (AHI) during sleep, providing comprehensive data. However, PSG requires specialists with sufficient time and space, resulting in a high cost. Although delayed detection of OSA can lead to life-threatening conditions, patients with OSA have to wait an average of 11.6 months before beginning medical therapy in Canada (8). Therefore, simple and practical methods are emerging to identify and classify patients at high-risk of OSA.

The need for a quick, user-friendly screening tool in clinics led to the development of the STOP-BANG questionnaire (SBQ), which includes eight dichotomous questions (9). This questionnaire provides superior reliability and accuracy in detecting OSA compared with the existing Berlin questionnaire or Epworth sleepiness scale (10). Because of the usefulness and convenience of the SBQ as well as the need for early detection of OSA, the SBQ has been implemented in the Korean National Health and Nutrition Examination Survey (KNHANES) since 2019.

Various studies have been conducted under the hypothesis that OSA affects auditory function. Numerous studies have evaluated the correlation between OSA and auditory dysfunction, but the results are inconsistent. Some studies have claimed a significant relationship between OSA and hearing, such as meaningful hearing loss at high frequencies or quantitative association with the severity of OSA (11–13). In contrast, other studies have found no relationship between OSA and hearing at all (14, 15). While some studies argued that the effect of OSA on hearing was due to recurrent hypoxemia affecting the cochlea (1), other studies have argued that the OSA affects the central auditory pathway, not the cochlea (14, 15). These diverse results are likely due to the small sample size of the studies, the different methods of defining OSA and hearing level, and the extent to which factors affecting hearing are adjusted. Therefore, this study aimed to predict the association between OSA assessed by the SBQ and hearing loss in a large cohort from the KNHANES.

## Materials and methods

### Study design and participants

This study was based on data from the KNHANES conducted by the Disease Control Headquarters to produce nationwide statistics to identify the health and nutritional statuses of Koreans. A cohort was surveyed between 2019 and 2020. The target population was Koreans aged 1 year or older, and samples were extracted using a two-stage stratified cluster sample extraction method, with survey locations and households serving as the first and second extraction units, respectively. This study included participants who had completed the SBQ, which was administered to those older than 40 years, and had undergone hearing assessment using pure-tone audiogram. This study was approved by the authors’ institutional review board (approval number: 2022-12-023).

### Clinical and laboratory measurements

Anthropometric, health-related variables, and biochemical measurements were included in the analysis. Body mass index (BMI) was calculated as weight in kilograms divided by height squared in meters. Neck circumference (NC) was assessed by measuring at the horizontal level of the seventh cervical vertebra. Waist circumference (WC) was assessed by measuring at the midpoint between the lowest rib and the anterior iliac crest in the standing position. Blood pressure (BP) was measured three times at the sitting position after resting for 5 min, and the average of the second and third results was used for analysis. High BP was defined as systolic BP ≥ 140 mmHg, diastolic BP ≥ 90 mmHg, or taking antihypertensive medication. The fasting blood glucose (FBG), glycosylated hemoglobin (HbA1c) level, triglyceride (TG), total cholesterol (TC), high-density lipoprotein cholesterol, and low-density lipoprotein cholesterol (LDL) were measured after overnight fasting. Diabetes was defined as FBG ≥ 126 mg/dL, diagnosis by a doctor, use of hypoglycemic agents or insulin injections, or HbA1c ≥ 6.5%. Hyperlipidemia was defined as TC ≥ 240 mg/dL or taking cholesterol-lowering drugs. Hypertriglyceridemia was defined as TG ≥ 200 mg/dL. Smoking status was classified into two groups: never smoker and former/current smoker. Participants who had ceased smoking at the time of the survey were considered former smokers, regardless of the duration of smoking cessation.

### OSA risk assessment

OSA risk was assessed by the SBQ (9), which contains eight dichotomous questions: loud snoring (S), day-time tiredness (T), observed apnea (O), high BP (P), BMI > 30 kg/m^2^ (B), age > 50 years (A), NC > 40 cm (N), and male sex (G). The low-risk group was defined as those who answered “yes” to 0–2 questions; the intermediate-risk group was defined as those who answered “yes” to 3–4 questions; the high-risk group was defined as those who answered “yes” to 5–8 questions or those who answered “yes” to 2 or more STOP questions with a BMI > 30 kg/m^2^, a NC > 40 cm, or male sex.

### Audiometric evaluation

The hearing threshold was evaluated by trained audiologists using an automatic audiometer (GSI SA-203; Entomed Diagnostics AB, Lena Nodin, Sweden) in a soundproof booth. The thresholds for 0.5, 1, 2, 4, and 8 kHz from both ears were available. Hearing thresholds were measured in each ear by pure-tone audiometry using an ascending/descending technique in 5 dB steps at frequencies of 0.5, 1, 2, 3, 4, and 8 kHz. The order of sound frequency was assigned randomly (16, 17). The pure-tone average (PTA) was defined as the mean values of the pure-tone thresholds at 0.5, 1, 2, and 4 kHz, whereas the high-frequency average was defined as the mean values at 4 and 8 kHz. We compared the PTA from each ear and selected the better hearing level in order to exclude those with pathologically damaged hearing, such as those with chronic otitis media, sudden hearing loss, etc.

### Statistical analysis

All statistical analyses were conducted using SPSS version 21 for Windows (IBM Corp., Armonk, NY, United States). The mean and standard deviation were used for descriptive statistics. The variables were compared among the low-, intermediate-, and high-risk groups using analysis of variance with Bonferroni post-hoc analysis and Pearson’s chi-squared test. When the variables were compared between two risk groups (e.g., low- vs. intermediate/high-risk groups), *t*-test and chi-squared test were used. Multiple linear regression predicted the association between hearing level and OSA-associated factors. A value of *p* < 0.05 was considered statistically significant.

## Results

### Study population

**Figure 1:**
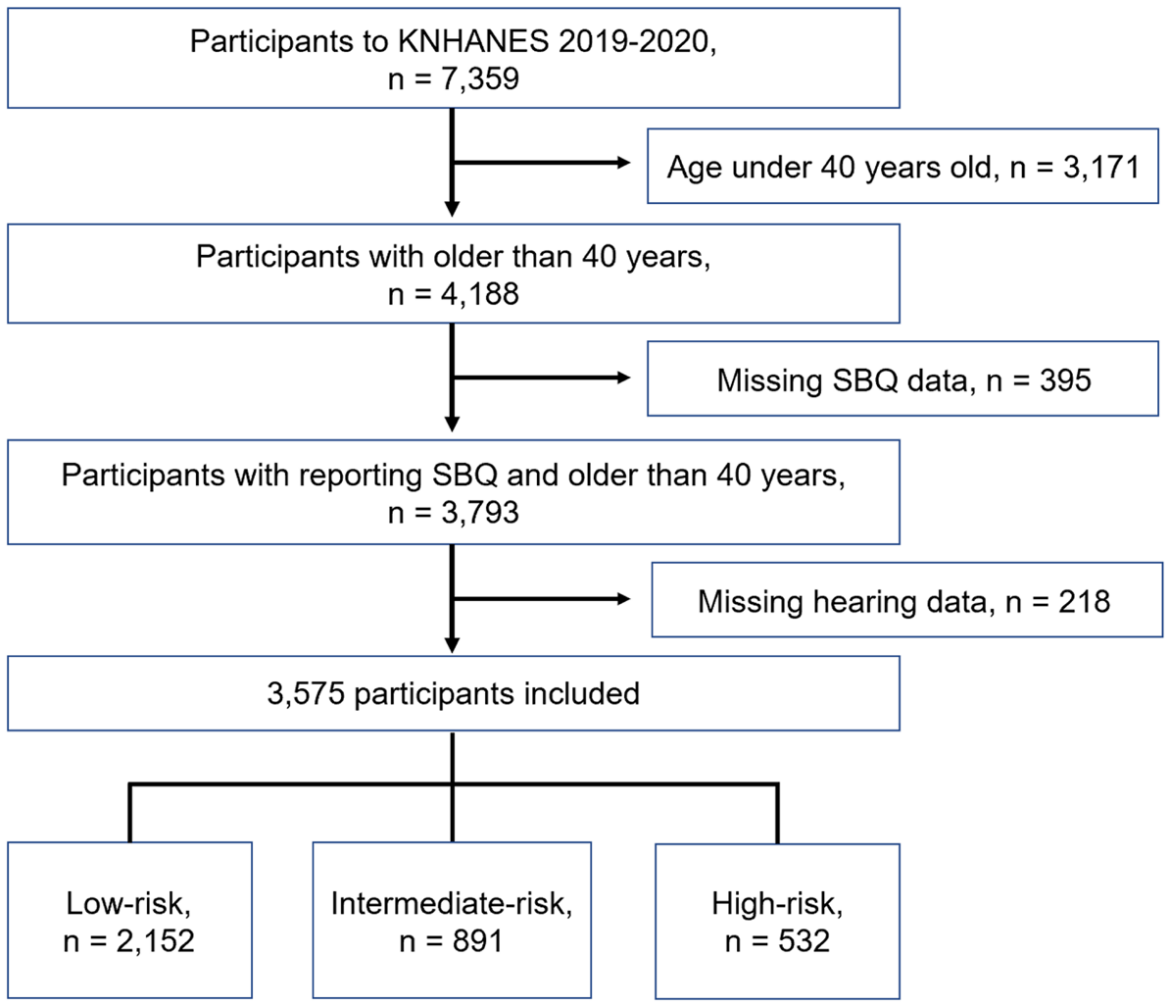
Schematic illustration of participant selection in the present study. KNHANES, Korean National Health and Nutrition Examination Survey; SBQ, STOP-BANG questionnaire.

A total of 7,359 participants, aged 1 year to 80 years, were included in the survey. A total of 4,188 participants were older than 40 years and were administered SBQ. Meanwhile, 395 and 218 participants were excluded due to missing SBQ and hearing assessment data, respectively. Finally, 3,575 participants were included in this study. The participants were grouped according to their STOP-BANG scores as follows: 2,152 participants (60.2%) were classified as having low risk, 891 (24.9%) were classified as having intermediate risk, and 532 (14.9%) were classified as having high risk (Figure 1). The results of the SBQ according to risk group are shown in Table 1. The higher the risk, the higher the proportion of most variables, except for age. The intermediate-risk group had a higher proportion of participants aged >50 years than the high-risk group.

**Table 1:** Variables of STOP-BANG questionnaire in each risk group.

	Total (n = 3,575)	Low risk (n = 2,152)	Intermediate risk (n = 891)	High risk (n = 532)	*p*-Value*	Intermediate/high risk (n = 1,423)	*p*-Value^#^
STOP-BANG score	2.3 ± 1.4	1.4 ± 0.7	3.1 ± 0.3	4.5 ± 1.0	<0.001	3.6 ± 0.9	<0.001
Loud snoring	710 (19.1%)	98 (4.6%)	248 (27.8%)	364 (68.3%)	<0.001	612 (43.0%)	<0.001
Day-time tiredness	1,052 (29.4%)	384 (17.8%)	352 (39.5%)	316 (59.3%)	<0.001	668 (46.9%)	<0.001
Observed apnea	319 (8.9%)	27 (1.3%)	70 (7.8%)	222 (41.7%)	<0.001	292 (20.5%)	<0.001
High blood pressure	1,469 (41.1%)	462 (21.5%)	617 (69.2%)	390 (73.2%)	Low vs. intermediate: <0.001 Low vs. high: <0.001 Intermediate vs. high: 0.252	1,007 (70.7%)	<0.001
Body mass index >30 kg/m^2^	152 (4.3%)	25 (1.2%)	44 (4.9%)	83 (15.6%)	<0.001	127 (8.9%)	<0.001
Age > 50 years	2,657 (74.3%)	1,406 (65.3%)	848 (95.1%)	403 (75.6%)	<0.001	1,251 (87.9%)	<0.001
Neck circumference > 40 cm	228 (6.4%)	11 (0.5%)	91 (10.2%)	126 (23.6%)	<0.001	217 (15.2%)	<0.001
Male sex	1,550 (43.4%)	541 (25.1%)	521 (58.4%)	488 (91.6%)	<0.001	1,009 (70.9%)	<0.001

^*^p-value: comparing three groups by one-way ANOVA with Bonferroni post-hoc analysis for a continuous variable and by chi-squared test for nominal variables. ^#^*p*-value: comparing two groups (low- vs. intermediate/high-risk groups) by an independent *t*-test for a continuous variable and by the chi-squared test for nominal variables. Continuous variable: STOP-BANG score. Nominal variables: Loud snoring, day-time tiredness, observed apnea, high blood pressure, body mass index, age, neck circumference and male sex. ANOVA, analysis of variance; STOP-BANG: S, loud snoring; T, day-time tiredness; O, observed apnea; P, high blood pressure; B, body mass index > 30 kg/m^2^; A, age > 50 years; N, neck circumference > 40 cm; G, male sex.

### Concordance between the SBQ and OSA diagnoses

The KNHANES included a question about whether the participants had been diagnosed with OSA by a doctor using PSG. The answers were analyzed as “yes,” “no,” and “do not know/no response.” A total of 22 participants answered “yes,” whereas 3,553 answered “no.” Of the 22 participants who had been diagnosed with OSA, 5 were low risk, 1 was intermediate risk, and 16 were high risk according to the SBQ.

### Comparison of OSA-associated factors according to the risk of OSA

The factors previously known to affect OSA were compared according to the risk of OSA (Table 2). The missing data for each factor was ≤2%, except for LDL (*n* = 506). When the variables were compared among the three groups, most showed significant differences. Systolic BP, FBG, HbA1c, presence of diabetes, TC, and presence of hyperlipidemia showed no differences between the intermediate- and high-risk groups, whereas these values in the intermediate/high-risk groups were significantly different from those in the low-risk group. Age was highest in the intermediate-risk group, and it was significantly different from that in the low- and high-risk groups; however, age did not differ between the low- and high-risk groups. LDL was highest in the low-risk group, and the difference was only significant between the low- and intermediate-risk groups. All variables showed significant differences between the low- and intermediate/high-risk groups.

**Table 2:** Comparison of OSA-associated factors according to the risk of OSA.

	Total (*n* = 3,575)	Low risk (*n* = 2,152)	Intermediate risk (*n* = 891)	High risk (*n* = 532)	*p*-value^*^	Intermediate/high risk (*n* = 1,423)	*p*-value^#^
Age (years)	59.5 ± 11.4	57.5 ± 11.5	64.8 ± 9.4	59.1 ± 10.8	<0.01	62.6 ± 10.3	<0.001
Neck circumference (cm)	35.8 ± 3.4	33.9 ± 2.8	36.3 ± 3.1	38.7 ± 2.5	<0.001	37.2 ± 3.1	<0.001
BMI (kg/m^2^)	24.3 ± 3.3	23.5 ± 3.0	25.0 ± 3.2	26.4 ± 3.7	<0.001	25.5 ± 3.4	<0.001
WC (cm)	85.8 ± 9.6	82.7 ± 8.8	88.9 ± 8.6	93.1 ± 8.9	<0.001	90.5 ± 9.0	<0.001
Smoking	19.8%	14.6%	50.2%	76.7%	<0.001	27.8%	<0.001
SBP (mmHg)	122.1 ± 16.4	118.2 ± 15.3	128.5 ± 16.7	127.5 ± 15.3	Low vs. intermediate: <0.001 Low vs. high: <0.001 Intermediate vs. high: 0.760	128.1 ± 16.2	<0.001
DBP (mmHg)	76.3 ± 9.8	74.9 ± 8.9	77.1 ± 10.1	80.9 ± 10.9	<0.001	78.5 ± 10.6	<0.001
FBG (mg/dL)	104.4 ± 23.5	101.3 ± 20.9	108.1 ± 24.4	111.0 ± 29.3	Low vs. intermediate: <0.001 Low vs. high: <0.001 Intermediate vs. high: 0.065	109.2 ± 26.3	<0.001
HbA1c (%)	6.0 ± 0.9	5.9 ± 0.9	6.2 ± 0.9	6.2 ± 1.03	Low vs. intermediate: <0.001 Low vs. high: <0.001 Intermediate vs. high: 0.250	6.2 ± 1.0	<0.001
DM	33.1%	29.1%	26.8%	29.3%	Low vs. intermediate: <0.001 Low vs. high: <0.001 Intermediate vs. high: 0.309	39.1%	<0.001
TC (mg/dL)	190.4 ± 40.4	195.3 ± 38.9	182.7 ± 41.5	183.3 ± 41.5	Low vs. intermediate: <0.001 Low vs. high: <0.001 Intermediate vs. high: 1.000	182.9 ± 41.5	<0.001
HDL (mg/dL)	50.8 ± 12.3	53.0 ± 12.5	48.7 ± 11.8	45.5 ± 10.0	<0.001	47.5 ± 11.3	<0.001
TG (mg/dL)	135.5 ± 111.5	123.5 ± 86.0	140.4 ± 123.7	176.0 ± 160.4	<0.001	153.7 ± 139.6	<0.001
LDL (n = 508)	111.9 ± 36.9	117.0 ± 38.4 (n = 240)	107.2 ± 34.4 (n = 142)	107.5 ± 35.5 (n = 126)	Low vs. intermediate: <0.05 Low vs. high: 0.059 Intermediate vs. high: 1.000	107.4 ± 34.9 (n = 268)	0.03
Hyperlipidermia	13.8%	11.0%	40.5%	36.6%	Low vs. intermediate: <0.001 Low vs. high: <0.001 Intermediate vs. high: 0.153	17.9%	<0.001
Hypertriglycemia	39.%	25.9%	14.9%	23.2%	<0.001	60.1%	<0.001

^*^p-value: comparing three groups by one-way ANOVA with Bonferroni post-hoc analysis for continuous variables and by chi-squared test for nominal variables. ^#^*p*-value: comparing two groups (low- vs. intermediate/high-risk groups) by an independent *t*-test for continuous variables and by chi-squared test for nominal variables. Continuous variables: age, neck circumference, BMI, WC, SBP, DBP, FBG, HbA1c, TC, HDL, TG and LDL. Nominal variables: smoking, DM, hyperlipidermina, and hypertriglycemia. ANOVA, analysis of variance; BMI, body mass index; WC, waist circumference; SBP, systolic blood pressure; DBP, diastolic blood pressure; FBG, fasting blood glucose; HbA1c, glycosylated hemoglobin; DM, diabetes mellitus; HDL, high-density lipoprotein cholesterol; OSA, obstructive sleep apnea; TC, total cholesterol; TG, triglyceride; LDL, low-density lipoprotein cholesterol.

### Hearing level according to the risk of OSA

**Figure 2:**
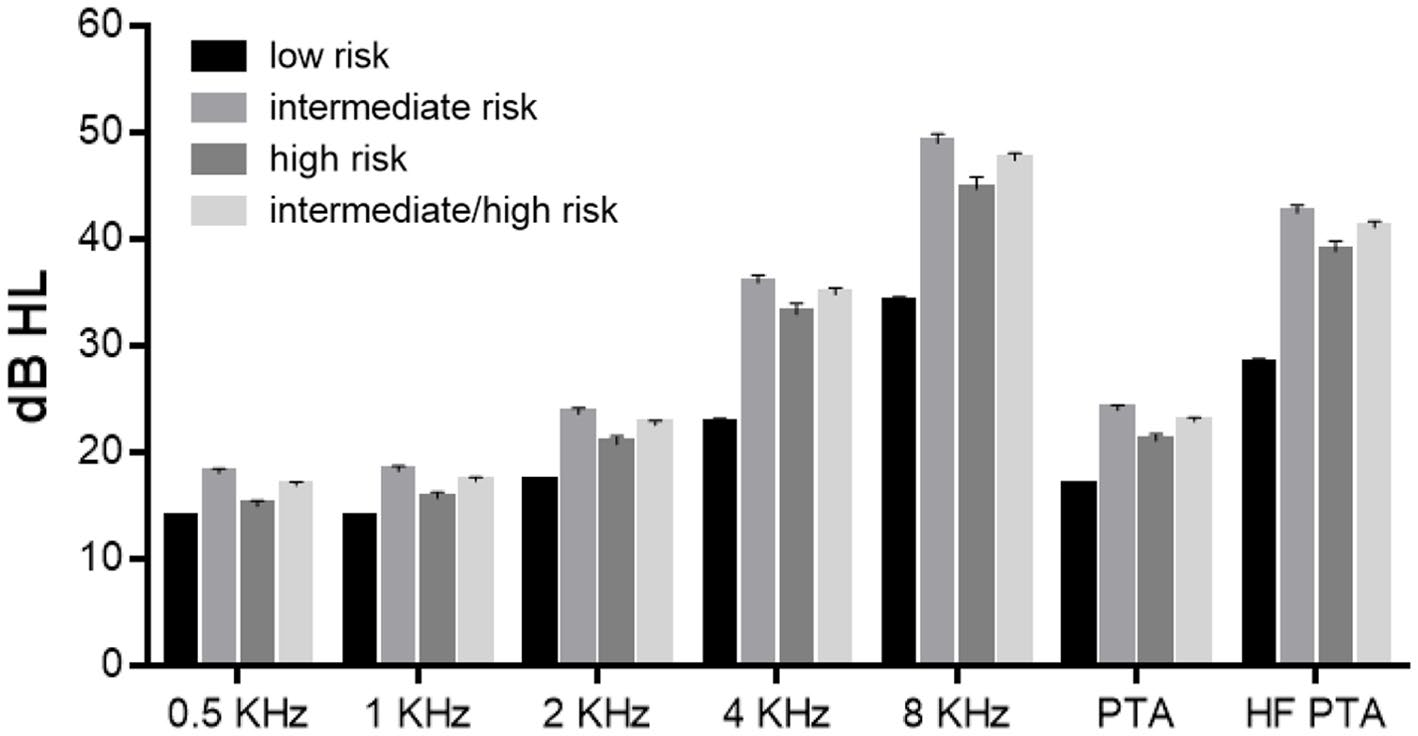
Hearing level according to the risk of obstructive sleep apnea (OSA). The hearing level differed significantly between the low- and intermediate-risk groups, the low- and high-risk groups, the low- and intermediate/high-risk groups, and the intermediate- and high-risk groups, except for the hearing level in the low- and high-risk groups at 500 Hz (*p* = 0.178).

Hearing level was analyzed according to the risk of OSA (Figure 2). The hearing level was worse in the intermediate-, high-, and intermediate/high-risk groups than that in the low-risk group. The differences in the hearing level were all significant, except for that in the low-risk group and that in the high-risk group at 500 Hz (*p* = 0.178). For all frequencies, the hearing level in the intermediate-risk group was worse than that in the high-risk group.

Multiple regression analysis was performed to identify whether any factor in the SBQ or any OSA-associated factor affected the hearing level. Among the 17 variables, age and sex significantly affected the hearing level. For PTA, the regression coefficient was 0.597 for age and 0.156 for sex. For high-frequency PTA, the regression coefficient was 0.648 for age and 0.268 for sex (Supplement 1). Because we aimed to determine the effect of OSA on hearing level, data adjusted for age and sex further analyzed. The data were adjusted by diving the total population into eight groups by age (40–50, 51–60, 61–70, and 71–80 years) and by sex. In all eight subgroups, no significant difference between age and sex was observed between the low- and intermediate/high-risk groups. Within the subgroups, the hearing levels according to the frequencies were compared between the low- and intermediate/high-risk group. Hearing level differences were found in a small proportion of participants (Supplement 2): at 8 kHz in 71–80 year-old males (low-risk group, 74.5 ± 18.3 dB, vs. intermediate/high-risk group, 68.5 ± 20.7 dB, *p* = 0.017), at 0.5 kHz in 51–60 year-old females (low-risk group, 11.4 ± 8.0 dB, vs. intermediate/high-risk group, 13.3 ± 9.3 dB, *p* = 0.027), and at 4 and 8 kHz in 71–80 year-old females (low-risk group, 37.7 ± 16.9 dB, vs. intermediate/high-risk group, 42.9 ± 17.2 dB, *p* = 0.005, and low-risk group, 60.8 ± 18.8 dB, vs. intermediate/high-risk group, 64.9 ± 17.7 dB, *p* = 0.034, respectively).

## Discussion

The present study investigated the relationship between OSA and hearing loss in a large cohort from the KNHANES. It included 3,575 participants and used the actual hearing level measured by frequency, rather than the pass/non-pass results of general health checkups.

In the present study, the risk of OSA was screened using the SBQ, which is a validated and easy-to-apply tool for screening OSA that requires careful interpretation. As the severity of OSA increases, the sensitivity of the SBQ increases. The probability of obtaining an SBQ score ≥ 3 has been reported to be 84% in patients with OSA (AHI > 5) and 100% in patients with severe OSA (AHI > 30) (9). However, among those with an SBQ score of 3, the probability of having OSA is 72%, whereas the probability of having severe OSA decreased to 13%. Even with an SBQ score of 8, the probability of having OSA is 86%, whereas that of having severe OSA is as low as 38% (18). In other words, among the general population whose OSA status is unknown, the SBQ can only predict the presence of OSA, but not the severity. Therefore, the results indicating low, intermediate, and high risks should not be misinterpreted as indications of the severity of OSA. In addition, the present study found that the known OSA-related factors showed significant quantitative differences in each group; however, these differences should not be interpreted as representing the severity of OSA.

We aimed to determine the concordance between the SBQ and OSA diagnoses; however, this analysis was not possible due to the small number of patients and lack of reliability in the test results. Among 3,575 participants, only 22 answered “yes” to whether they had been diagnosed with OSA by a doctor using a PSG. The calculated probability of obtaining an SBQ score ≥ 3 in patients with OSA is 77%, which is slightly lower than that suggested by its developers. Interestingly, the remaining 3,553 participants answered “no” to this question, although the answer options were “yes,” “no,” and “do not know/no response.” It is highly likely that the answers “no” and “do not know/no response” are interpreted interchangeably by the participants. However, the fact that only 22 participants answered “yes” to this question underscores the difficulty in accurately diagnosing OSA because either the participants did not suspect OSA themselves or the process leading to a PSG is difficult. These results once again highlight the importance of an easily accessible OSA questionnaire.

The present study found that the hearing level of the intermediate/high-risk group was worse than that of the low-risk group. However, when the factors that affect hearing, such as age and sex, were adjusted, no significant difference was observed in hearing level between the two groups. This result differs greatly from previous studies. Similar to this study, a study that evaluated the relationship between SBQ scores and hearing level in 794 Chinese males aged 40–65 years reported that the hearing level of the intermediate/high-risk group was worse, even after adjusting for age, BMI, and cardiovascular risk (19). However, the study used pass/fail results of hearing screening centered at 25 dB, which could be seen as relatively less accurate in evaluating the hearing level than the present study. A systematic meta-analysis published in 2022 also concluded that OSA and hearing loss were significantly correlated and that hearing loss was affected by the severity of OSA (13). They argued that age was adjusted because most of the papers they included were age-matching studies; however, only 11 of the 20 papers they included were age-matched. Therefore, we believe that the meta-analysis cannot claim that its data are age-matched, as only a subset of its data are age-matched and subgroup analyses may be misleading. Meanwhile, Hwang et al. and İriz et al. did not find any differences in PTA between patients with and without OSA after adjusting for age, sex, and other variables (14, 15).

The effect of OSA on hearing has not been clearly defined, but most studies have agreed that the main pathology of hearing loss in patients with OSA is cochlear ischemia due to chronic intermittent hypoxemia (20, 21). Because this pathophysiology does not occur suddenly like noise exposure or infection, damage to the cochlea likely occurs gradually over a long period of time and does not recover. A previous study investigated whether hearing was restored when OSA was treated using CPAP and found that hearing was unchanged (13), confirming that damage to the cochlea occurs over a long period of time and the damage is irreversible. Considering that hearing loss in OSA is a long-term phenomenon, it would be difficult to predict the effect of OSA on hearing using the current PSG parameters. The parameters are a result of measurements taken at a given time in a patient with OSA. In other words, to determine the actual effect of OSA on hearing, we should not only examine the frequency of apnea or hypopnea (AHI) or the lowest oxygen saturation level (lowest SaO_2_) at a given time but also investigate the duration of exposure to hypoxemia. This will also explain the paradoxical relationship between PSG results and hearing in the older adult. As age increases, the frequency of desaturation events (O_2_ nadir) becomes lower, and the lowest saturation level increases, which means that the hypoxic state is less induced in the older adult with OSA than in middle-aged adults, suggesting that the older adult will have less damage from hypoxia (22). Nevertheless, that hearing level deteriorated with increasing age supports the fact that long-term hypoxia has a greater effect on hearing than fragmentary hypoxia. Therefore, the fact that the previous studies have shown so many different results and that age has a great effect on hearing in most studies, including the present study, could be accepted to some extent. Moreover, determining the duration of OSA is even more difficult to determine than the diagnosis of OSA. Thus, no study has investigated the relationship between the duration of OSA and hearing. Hence, a long-term comparison of the hearing level of patients with and without treatment after diagnosis of OSA is warranted.

The present study found no significant correlation between the presence of OSA and hearing level in a large cohort after adjusting for other factors that affect hearing. However, these results should be interpreted carefully. First, because hearing loss due to OSA is a long-term event, the relationship between OSA and hearing cannot be simply evaluated by the presence or absence of OSA using the current cross-sectional data. Moreover, evaluating the effect of OSA on hearing only with the results of pure-tone audiometry, which only measures the audible range, has limitations. For example, there could be differences in frequencies above 8 kHz that are more vulnerable to damage, in otoacoustic emission test that measures cochlear function, or in central auditory functions. Nevertheless, the present study confirmed the need to evaluate the effects of OSA on hearing loss in greater detail and with a longer duration.

## Data availability statement

The original contributions presented in the study are included in the article/Supplementary material, further inquiries can be directed to the corresponding author.

## Ethics statement

The studies involving human participants were reviewed and approved by the authors’ institutional review board (Inje University College of Medicine, approval number: 2022-12-023). Written informed consent for participation was not required for this study in accordance with the national legislation and the institutional requirements.

## Author contributions

JML and HJL: conceptualization, methodology, project administration, visualization, and writing—review and editing. JML: data curation and writing—original draft. HJL: formal analysis. All authors contributed to the article and approved the submitted version.

## Funding

This work was supported by the National Research Foundation of Korea (NRF) grants funded by the Korea government (MSIT) to JML (no. 2022R1F1A1071824). This work was also supported by the Ministry of Education of the Republic of Korea and the National Research Foundation of Korea (NRF-RS-2023-00210073).

## Conflict of interest

The authors declare that the research was conducted in the absence of any commercial or financial relationships that could be construed as a potential conflict of interest.

## Publisher’s note

All claims expressed in this article are solely those of the authors and do not necessarily represent those of their affiliated organizations, or those of the publisher, the editors and the reviewers. Any product that may be evaluated in this article, or claim that may be made by its manufacturer, is not guaranteed or endorsed by the publisher.
